# Humoral and T-cell-mediated responses to an insect-specific flavivirus-based Zika virus vaccine candidate

**DOI:** 10.1371/journal.ppat.1012566

**Published:** 2024-10-10

**Authors:** Danielle L. Porier, Awadalkareem Adam, Lin Kang, Pawel Michalak, Juselyn Tupik, Matthew A. Santos, Manette Tanelus, Krisangel López, Dawn I. Auguste, Christy Lee, Irving C. Allen, Tian Wang, Albert J. Auguste

**Affiliations:** 1 Department of Entomology, Virginia Tech, Blacksburg, Virginia, United States of America; 2 Department of Microbiology & Immunology, University of Texas Medical Branch, Galveston, Texas, United States of America; 3 Department of Biomedical Research, Edward Via College of Osteopathic Medicine, Monroe, Louisiana, United States of America; 4 Department of Biomedical Sciences and Pathobiology, Virginia-Maryland College of Veterinary Medicine, Blacksburg, Virginia, United States of America; 5 College of Pharmacy, University of Louisiana Monroe, Monroe, Louisiana, United States of America; 6 Center for One Health Research, Virginia-Maryland College of Veterinary Medicine, Blacksburg, Virginia, United States of America; 7 Institute of Evolution, University of Haifa, Haifa, Israel; 8 Center for Emerging, Zoonotic, and Arthropod-borne Pathogens, Virginia Tech, Blacksburg, Virginia, United States of America; University of California San Francisco, UNITED STATES OF AMERICA

## Abstract

Flaviviruses represent a significant global health threat and relatively few licensed vaccines exist to protect against them. Insect-specific flaviviruses (ISFVs) are incapable of replication in humans and have emerged as a novel and promising tool for flavivirus vaccine development. ISFV-based flavivirus vaccines have shown exceptional safety, immunogenicity, and efficacy, however, a detailed assessment of the correlates of protection and immune responses induced by these vaccines are still needed for vaccine optimization. Here, we explore the mechanisms of protective immunity induced by a previously created pre-clinical Zika virus (ZIKV) vaccine candidate, called Aripo/Zika (ARPV/ZIKV). In brief, immunocompromised IFN-αβR^-/-^ mice passively immunized with ARPV/ZIKV immune sera experienced protection after lethal ZIKV challenge, although this protection was incomplete. ARPV/ZIKV-vaccinated IFN-αβR^-/-^ mice depleted of CD4^+^ or CD8^+^ T-cells at the time of ZIKV challenge showed no morbidity or mortality. However, the adoptive transfer of ARPV/ZIKV-primed T-cells into recipient IFN-αβR^-/-^ mice resulted in a two-day median increase in survival time compared to controls. Altogether, these results suggest that ARPV/ZIKV-induced protection is primarily mediated by neutralizing antibodies at the time of challenge and that T-cells may play a comparatively minor but cumulative role in the protection observed. Lastly, ARPV/ZIKV-vaccinated Tcra KO mice, which are deficient in T-cell responses, experienced significant mortality post-challenge. These results suggest that ARPV/ZIKV-induced cell-mediated responses are critical for development of protective immune responses at vaccination. Despite the strong focus on neutralizing antibody responses to novel flavivirus vaccine candidates, these results suggest that cell-mediated responses induced by ISFV-based vaccines remain important to overall protective responses.

## Author summary

Conventional vaccine development platforms may involve trade-offs between vaccine safety and immunogenicity, but insect-specific viruses have recently emerged as a promising platform to overcome this challenge. We previously developed a preclinical Zika virus (ZIKV) vaccine candidate named Aripo/Zika virus (ARPV/ZIKV) based on a novel ISFV called Aripo virus (ARPV). Our previous studies demonstrated the high degree of safety as well as the single dose efficacy of ARPV/ZIKV. Here, we begin to elucidate the mechanisms of protection for this vaccine candidate. We demonstrate the dominant role of neutralizing antibodies in providing protection post-challenge, but also the importance of ARPV/ZIKV-induced T-cell responses in the priming phase of immunity. Overall, even high efficacy ISFV-based vaccine candidates such as ARPV/ZIKV may benefit from adjuvants or optimization strategies to increase protective T-cell responses. However, ARPV/ZIKV remains a promising ZIKV vaccine candidate, and contributes to the rapidly growing body of work that supports the potential of ISFVs as a new tool for protecting the health of the millions of people currently at risk of infection.

## 1. Introduction

The *Flavivirus* genus has a near-global distribution, and its members pose a significant threat to human and animal health. Flaviviruses continue to emerge worldwide, causing significant morbidity and mortality [1–4]. In particular, Zika virus (ZIKV) caused outbreaks throughout the South Pacific until 1997 [5,6], before causing an explosive epidemic in the Americas that resulted in over half a million suspected cases in South America between 2015 and 2017 [7]. Between 2010 and 2019, ZIKV caused an average loss of over 44,000 disability-adjusted life years [8], establishing its importance as an arboviral pathogen.

Despite the global health burden of ZIKV, licensed vaccines for humans remain elusive. In the decade since ZIKV’s emergence in the Americas, no vaccine candidates have progressed to Phase III clinical trials, nor been approved for use in humans. Currently, there are two candidates in active Phase II clinical trials: an mRNA vaccine developed by Moderna (NCT04917861), and an inactivated vaccine developed by Takeda (NCT05469802). Previously, we created a cell culture-based live recombinant Zika vaccine candidate (called ARPV/ZIKV) containing the genes for key antigenic Zika proteins (precursor membrane (prM) and envelope (E) on an Aripo virus (ARPV) backbone [9]. ARPV is a recently discovered insect-specific flavivirus (ISFV) that was isolated from *Psorophora albipes* mosquitoes in Trinidad [10]. Insect-specific viruses (ISVs), including ISFVs, are incapable of replication within vertebrate hosts, and within the past decade have emerged as a promising tool for controlling vertebrate-pathogenic viruses. ISVs have been used to create several vaccine candidates against both alphaviruses and flaviviruses [9,11–17]. These live recombinant vaccines are extremely safe due to the natural vertebrate host-restriction conferred to them by the insect-specific virus backbone, yet still retain significant immunogenicity, unlike traditional inactivated vaccines that may ultimately require several doses to achieve the desired efficacy.

We demonstrated that a single dose of unadjuvanted ARPV/ZIKV completely protected mice from weight loss, death, viremia, and *in utero* transmission after ZIKV challenge [9]. ARPV/ZIKV’s high degree of efficacy is likely, in part, attributable to its rapid and robust induction of high neutralizing antibody (nAb) titers against ZIKV [9]. However, little is known about the immune responses and correlates of protection to ISFV-based vaccines (such as ARPV/ZIKV), or whether these responses vary significantly to those observed during primary flavivirus infection.

During ZIKV infection, humoral nAb responses are critical for controlling viremia and mediating protection [18,19]. CD4^+^ T-cells are also important for protection against ZIKV-induced disease, and especially for their role in generating ZIKV-specific humoral responses [20], but they may also play a limited role in ZIKV clearance [20,21]. Murine and non-human primate studies have also demonstrated a role for CD8^+^ T-cell responses in protecting against viral dissemination and persistence in tissues, although they may not be critical for controlling viremia [19,21–23]. Lastly, cross-reactivity among flavivirus antibodies at sub-neutralizing levels also raises concerns for antibody-dependent enhancement of disease (ADE) [18]. However, robust T-cell responses may help alleviate concerns about inducing dengue virus ADE after ZIKV vaccination [18,19].

Insect-specific virus-based vaccines, especially ISFV-based vaccines, are a relatively new field of study, and a better understanding of their correlates of protection are needed. However, the role of T-cell and nAb responses in ARPV/ZIKV-induced protection against Zika disease has not been elucidated. Here, we use ARPV/ZIKV as a model to examine the roles of humoral and cell-mediated adaptive immunity induced by ISFV-based vaccines in murine models. Through the application of passive transfer, adoptive transfer, and T-cell-depletion studies in mice, we found that ARPV/ZIKV-induced protection is primarily mediated by nAbs. However, vaccination of muMt^-^ and Tcra KO mice showed that T-cell responses are critical for the development of immunity post ARPV/ZIKV vaccination. Overall, these data indicate that optimal responses to ISFV-based vaccines will likely require stimulation of both cell-mediated and humoral immunity.

## 2. Materials and methods

### 2.1. Ethics statement

All experimental animal procedures were approved by Virginia Tech Institutional Animal Care and Use Committee and conform to regulatory standards.

### 2.2. Cell lines, viruses, and quantification

VERO 76 (Cat# CRL-1587, RRID: CVCL_0603) and C6/36 (Cat# CRL-1660, RRID: CVCL_Z230) cells from ATCC (Manassas, VA, USA) were maintained according to ATCC guidelines. ARPV isolation and ARPV/ZIKV development was previously described [9,10]. All viruses were maintained in C6/36 cells. Viral RNA was extracted using QIAmp Viral RNA Mini kits (QIAGEN, Venlo, Netherlands), according to the manufacturer’s instructions. Viral quantification was performed using reverse transcription quantitative PCR (RT-qPCR) (described [9]) or by plaque assay on VERO 76 cells. ZIKV-specific nAb titers in blood were quantified by plaque reduction neutralization test (PRNT_50_ or PRNT_80_) on VERO 76 cells against ZIKV strain PRVABC59 as previously described [24], except that incubations were maintained for 4 days before fixation. The lower limit of detection for the PRNT assays was the 20-fold dilution and the upper limit of detection was the 640-fold dilution.

### 2.3. Animal experiments

ARPV/ZIKV and ARPV inoculums were prepared as previously described [9] and diluted with phosphate buffered saline (PBS) to achieve the desired dose. All mouse strains were purchased from Jackson Laboratory (Bar Harbor, ME, USA), and include: C57BL/6J mice (strain #000664, RRID: IMSR_JAX:000664), Rag1 KO mice (strain #002216, RRID: IMSR_JAX:002216), muMt^-^ mice (strain #002288, RRID: IMSR_JAX:002288), and Tcra KO mice (strain #002116, RRID: IMSR_JAX:002116). IFN-αβR^-/-^ mice (strain #032045-JAX, RRID: MMRRC_032045-JAX) were also purchased from Jackson Laboratory and subsequently bred in-house.

During challenge studies, four-week-old mice were divided randomly into groups prior to subcutaneous (s.c.) administration of optimal, maximal doses of 10^12^ genome copies (GC) of ARPV/ZIKV, 10^11^ GC ARPV (vaccine backbone control), 10^8^ GC of mouse-attenuated ZIKV PRVABC59 (immunogenic positive control), or sham-vaccinated with PBS (naïve control). The ARPV/ZIKV dose was selected based on previously reported dose de-escalation studies that showed 10^12^ GC was the optimal dose for complete protection against ZIKV challenge [25]. Although ZIKV PRVABC59 was administered at a lower titer, this live mouse-attenuated virus was administered at a dose known to be highly immunogenic and fully protective based on previous studies [9,25]. At 26 or 28 days post-vaccination (dpv), blood was collected from the retro-orbital sinus. The presence of ZIKV-specific nAb in serum prior to challenge was assessed by PRNT.

A lethal dose of low-passage ZIKV DakAr D 41524, which is heterologous to the ZIKV strain used to develop ARPV/ZIKV [9], was administered s.c. at challenge (10^5^ plaque-forming units (pfu); 10^6^ GC). In the studies below, healthy control mice (unchallenged mouse group) that were initially immunized with PBS were administered PBS rather than ZIKV at challenge. Sham-vaccinated control mice were initially immunized with PBS-diluent and received the full ZIKV challenge dose. Mice were monitored daily for clinical signs of disease post-challenge. Blood was collected for up to 4 days post-challenge (dpc) to quantify viremia. Mice were euthanized after losing 20% or more of their original body weight, or upon demonstrating severe clinical symptoms such as lethargy, hunched posture, paralysis, or unresponsiveness.

### 2.4. Passive transfer of immune sera

Donor C57BL/6J mice were vaccinated as described above. Blood was collected 28 dpv for evaluation of ZIKV-specific nAb titers as described above. At 37 dpv, mice (n = 10–15) were euthanized and sera collected via cardiac puncture. Sera were pooled according to immunization group and ZIKV-specific nAb titers were assessed by PRNT for each pool. Pooled sera were transferred via intraperitoneal (i.p.) injection into 8-9-week-old mixed gender IFN-αβR^-/-^ mice (n = 7; 340 μL serum per mouse). Sham and unchallenged IFN-αβR^-/-^ mice received undiluted serum from mice that were initially immunized with PBS. IFN-αβR^-/-^ mice in the ARPV group and ZIKV group received undiluted serum from ARPV-immunized or ZIKV-immunized mice, respectively. IFN-αβR^-/-^ mice received ARPV/ZIKV serum in one of three different doses: (1) undiluted ARPV/ZIKV immune serum (high dose), (2) ARPV/ZIKV immune serum diluted 1:2 with PBS just prior to transfer (medium dose), or (3) ARPV/ZIKV immune serum diluted 1:5 (low dose). One day after transfer, sera were collected from IFN-αβR^-/-^ mice to quantify circulating nAb titers. Two days post-transfer, IFN-αβR^-/-^ mice were challenged as described above, except for the unchallenged mouse group which received PBS at challenge. Mice were monitored and viremia determined as described above.

### 2.5. T-cell depletion

Mixed gender IFN-αβR^-/-^ or C57BL/6J mice were vaccinated and challenged as described above. Monoclonal antibodies were purchased from Bio X Cell (Lebanon, NH, USA) and include: anti-CD4 clone GK1.5 (Cat# BE0003-1, RRID: AB_1107636), anti-CD8α clone 2.43 (Cat# BE0061, RRID: AB_1125541), and IgG2b isotype control clone LTF-2 (Cat# BE0090, RRID: AB_1107780). On -3, -1, +1, +3, and +6 dpc, mice in each immunization group received either 100 μg anti-CD4^+^ antibody, 250 μg anti-CD8^+^ antibody, both, 100 μg isotype antibody, or an equivalent volume of PBS (no depletion group) by i.p. injection (n = 4–7). C57BL/6J mice were also administered anti-mouse IFNAR-1 monoclonal antibody clone MAR1-5A3 (Leinco Technologies, Fenton, MO, USA; Cat# I-1188, RRID: AB_2830518) to transiently inhibit Type I IFN at the time of ZIKV challenge, as previously described [9]. Mice were challenged 30 dpv (i.e., 0 dpc). A group of age-matched, mixed gender mice (n = 2 per depletion type) were euthanized immediately prior to challenge and bled to confirm the efficiency of T-cell depletion. Blood was collected in BD Microtainer K_2_EDTA tubes (BD Biosciences, San Jose, CA, USA).

Lymphopure density gradient medium (BioLegend, San Diego, CA, USA) was used to separate lymphocytes as previously described [26]. Cells were resuspended in 3 mL TheraPEAK ACK Lysing Buffer (1X) (Lonza, Basel, Switzerland). After a 2-minute incubation, 10 mL PBS was added. Cells were re-suspended in flow cytometry buffer and incubated with TruStain FeX PLUS anti-mouse CD16/32 (BioLegend) (0.25 μg per 10^6^ cells in 100 μL) for 5 minutes on ice. Cells were stained with either BD Pharmingen APC Rat Anti-Mouse CD8α antibody (BD Biosciences Cat# 553035, RRID: ab_398527) or BD Horizon BV605 Rat Anti-Mouse CD4 antibody (BD Biosciences Cat# 563151, RRID: AB_2687549), at a final concentration of 1:1000 or 1:200 respectively. T-cell populations were measured on a FACSAria Fusion flow cytometer. After confirming depletion of CD8^+^ or CD4^+^ T-cells via flow cytometry, mice were challenged, and disease outcomes measured as described above.

### 2.6. Adoptive transfer of T-cells

C57BL/6J mice were vaccinated as described above. Spleens were harvested from mice 30 dpv and processed to collect viable splenocytes in a single cell suspension. Single-cell suspensions of CD4^+^ or CD8^+^ T-cells were prepared from spleens by negative selection using magnetic beads (Miltenyi Biotech, Bergisch Gladbach, Germany) following the manufacturer’s recommendations. Naive 6-week-old IFN-αβR^-/-^ mice (n = 4–9) received 5 × 10^6^ cells (i.p.) 24 hours prior to challenge. The mock group received T-cells isolated from naïve mice and the “no transfer” group received an equivalent volume of PBS diluent. Mice were challenged and disease outcomes measured as described above.

### 2.7. Vaccination of Tcra KO, Rag1 KO, and muMt^-^ mice

Tcra KO, Rag1 KO, and muMt^-^ mice were vaccinated (n = 6), challenged 30 dpv, monitored, and assessed for disease as described above. Anti-mouse IFNAR-1 monoclonal antibody (clone MAR1-5A3, Leinco Technologies) was used to transiently deplete Type I IFN during challenge as previously described [9].

### 2.8. Statistical analysis

GraphPad Prism (v9.0) was used for all analyses. Data distribution and variance were evaluated for normality and normalized by log10 transformation as needed. Log-rank (Mantel–Cox) tests were performed on Kaplan–Meier survival curves to evaluate statistical significance. PRNT and weight change data were evaluated for significance by one-way ANOVA at each day, and viremia data were evaluated by two-way ANOVA. Multiple comparisons among groups were performed ad hoc using Tukey’s test. Data in figures represent mean ± standard deviation (SD). Statistical results are noted as not significant (ns), *p* ≤ 0.033 (*), *p* ≤ 0.002 (**), *p* ≤ 0.0002 (***), or *p* ≤ 0.0001 (****).

### 2.9. Resource availability

Further information regarding requests for resources, materials, or reagents should be directed to and will be fulfilled by the corresponding author, Albert J. Auguste. This study did not generate unique reagents. The mouse strain used for this research project, B6.129S2-*Ifnar1*^tm1Agt^/Mmjax, RRID:MMRRC 032045-JAX, was obtained from the Mutant Mouse Resource and Research Center (MMRRC) at The Jackson Laboratory, an NIH-funded strain repository, and was donated to the MMRRC by Michel Aguet, Ph.D., Swiss Institute for Experimental Cancer Research.

## 3. Results

### 3.1. Passive transfer of neutralizing antibodies derived from ARPV/ZIKV immunized mice did not provide complete protection from a lethal ZIKV challenge

**Fig 1:**
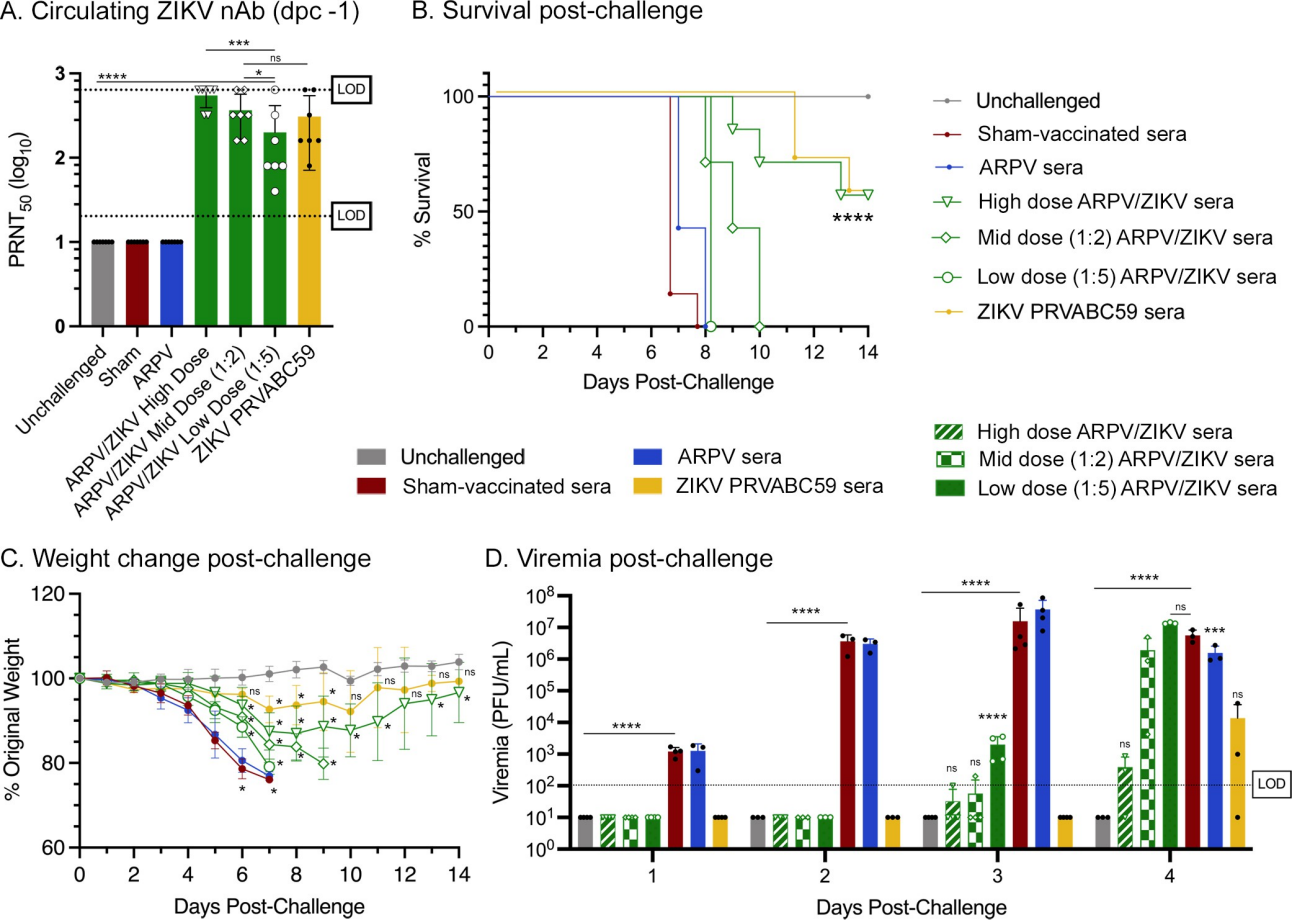
Passive transfer of ARPV/ZIKV-induced antibodies partially protects naïve mice. Four-week-old C57BL/6J mice were vaccinated as previously described. At 30 days post-vaccination, sera were pooled according to immunization group and injected into naïve 8–9 week-old IFN-αβR^-/-^ mice. (a) One day prior to challenge, mice were bled to quantify the amount of ZIKV-specific nAb circulating in the blood. Dotted lines indicate the lower 20-fold and upper 640-fold limits of detection (LOD). Mice were challenged with ZIKV, except for the unchallenged group. Mice were then monitored for (b) survival and (c) weight change post-challenge. (d) Sera were collected for 1–4 days post-challenge (dpc) to quantify viremia. LOD is 100 pfu/mL. (a, d) Columns represent mean values, and symbols represent individual data points. (c) Symbols represent mean values. Error bars indicate SD of the mean. Asterisks indicate significance compared to healthy mice (unchallenged controls), unless otherwise indicated: not significant (ns), *p* ≤ 0.033 (*), *p* ≤ 0.0002 (***), *p* ≤ 0.0001 (****).

Given the prominence of nAb responses as a critical correlate of protection during flavivirus infection [27–29] and prior studies that demonstrated ARPV/ZIKV’s ability to induce exceptionally high nAb titers [9], we hypothesized that ARPV/ZIKV-induced nAb responses alone may be sufficient to protect mice from ZIKV disease. To investigate this, APRV/ZIKV immune serum was transferred from vaccinated donor C57BL/6J mice to naïve recipient IFN-αβR^-/-^ mice pre-challenge (n = 7 recipient mice). One day pre-challenge, naïve mice that received a high, medium, or low dose of ARPV/ZIKV immune serum had circulating ZIKV-specific nAb titers of >640 PRNT_50_, 365 PRNT_50_, and 200 PRNT_50_, respectively. Mice that received undiluted ZIKV immune serum had titers of 308 PRNT_50_ (Fig 1A). Mice that received undiluted ZIKV immune serum showed the lowest mortality and weight loss (Fig 1B and 1C). These mice also had the lowest levels of viremia on days 1–3 post-challenge (Fig 1D). Mice that received sera from ARPV-immunized and sham-vaccinated mice, and the low dose of ARPV/ZIKV immune serum experienced 100% mortality by 8 dpc whereas mice that received a high dose of ARPV/ZIKV serum or ZIKV serum experienced 43% mortality by 14 dpc (Fig 1B). Despite this similarity in overall mortality, mice that received a high dose of ARPV/ZIKV immune serum demonstrated significantly more weight loss 8 dpc than mice that received ZIKV immune serum (Fig 1C), even though ARPV/ZIKV high dose mice presented higher nAb titers (Fig 1A). Overall, we observed that passive immunity from ARPV/ZIKV immune sera did not appear to confer complete protection during challenge. Additionally, comparative results between groups that received ARPV/ZIKV and ZIKV immune sera led to the hypothesis that differences in protection was not driven by differences in nAb titers alone.

### 3.2. CD4^+^ and CD8^+^ T-cells induced by ARPV/ZIKV vaccination may play a cumulative role in mediating protection after ZIKV challenge

**Fig 2:**
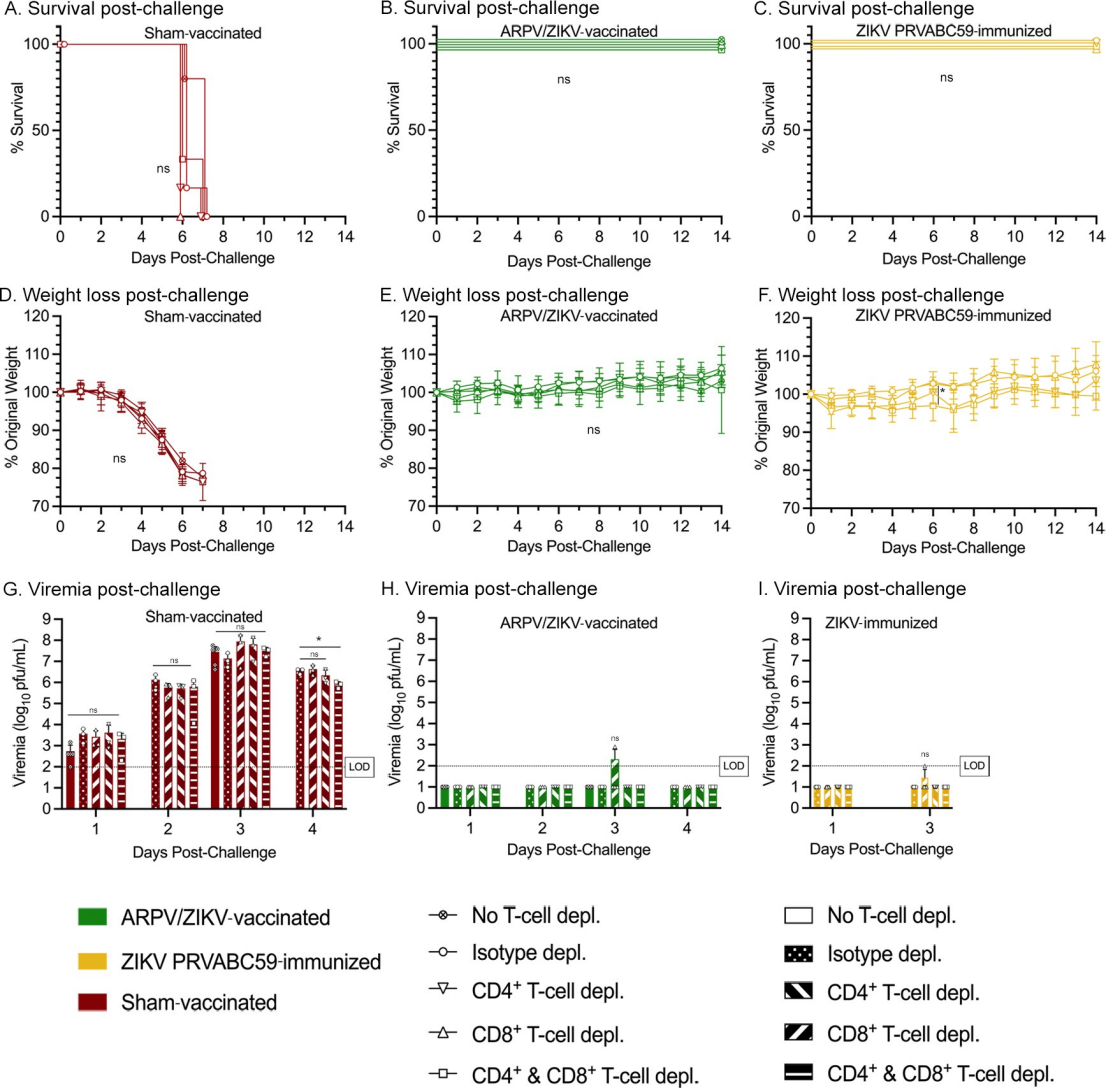
Depletion of T-cells in ARPV/ZIKV-vaccinated mice at challenge show neutralizing antibodies offer complete protection from ZIKV challenge. Four-week-old IFN-αβR^-/-^ mice were vaccinated as previously described. Beginning 27 days post-vaccination (dpv), T-cell depleting antibodies were administered according to depletion group (anti-CD4^+^, anti-CD8^+^, both, or isotype depletion). Mice were challenged with ZIKV at 30 dpv. Mice were monitored for (a-c) survival and (d-f) weight change post-challenge. (g-i) Sera were collected for 1–4 days post-challenge to quantify viremia. LOD is 100 pfu/mL. (d-f) Symbols represent mean values. (g-i) Columns represent mean values, and symbols represent individual data points. Error bars indicate SD of the mean. Asterisks indicate significance compared to isotype depleted control mice, unless otherwise indicated: not significant (ns), *p* ≤ 0.033 (*).

**Fig 3:**
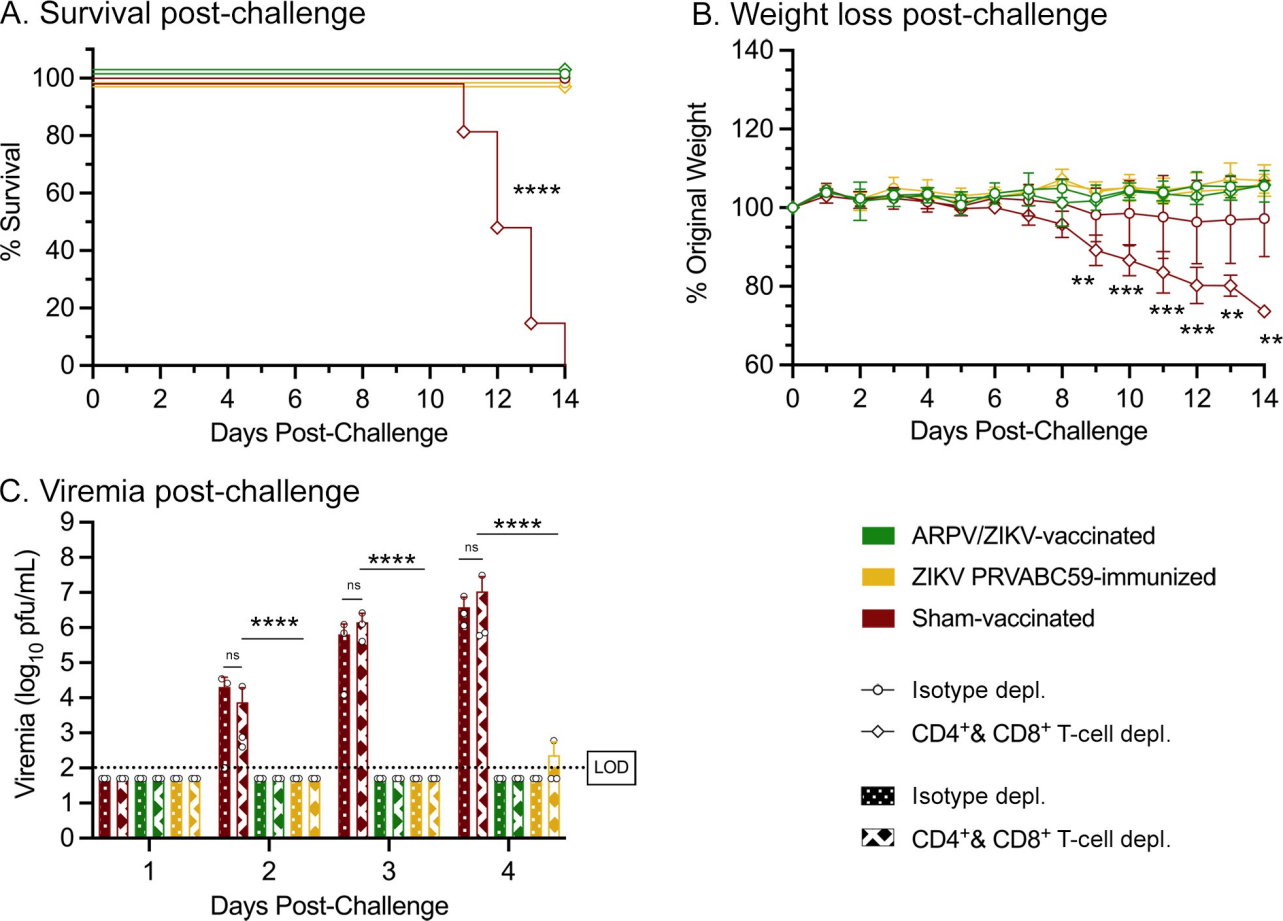
Depletion of T-cells in ARPV/ZIKV-vaccinated immunocompetent mice at challenge did not significantly impact clinical outcomes. Four-week-old C57BL/6J mice were vaccinated as previously described. Regimens of *in vivo* T-cell depletion monoclonal antibody were administered according to depletion group (anti-CD4^+^, anti-CD8^+^, both, or isotype depletion). At 30 days post-vaccination, mice were challenged with ZIKV. Mice were then monitored for (a) survival and (b) weight change post-challenge. (c) Sera were collected for 1–4 days post-challenge. LOD is 100 pfu/mL. (b) Symbols represent mean values. (c) Columns represent mean values, and symbols represent individual data points. Error bars indicate SD of the mean. Asterisks indicate significance compared to isotype depleted control mice, unless otherwise indicated: not significant (ns), *p* ≤ 0.002 (**), *p* ≤ 0.0002 (***), *p* ≤ 0.0001 (****).

To determine if the incomplete protection observed after passive transfer of ARPV/ZIKV immune sera might be due to the absence of primed T-cell responses during challenge and/or the lower circulating nAb titers compared to vaccinated mice, vaccinated IFN-αβR^-/-^ mice (nAb titers post-vaccination shown in S1 Fig; n = 4–7) were treated with anti-CD4^+^ and/or anti-CD8^+^ depleting antibodies immediately prior to challenge. There were no significant differences in survival, weight loss, or viremia between depletion treatments within ARPV/ZIKV-vaccinated or ZIKV-immunized mice (Fig 2), except for the ZIKV-immunized anti-CD4^+^ and -CD8^+^ group at 6 dpi (Fig 2F). Unchallenged and ARPV-immunized mice showed no significant differences in survival, weight loss, or viremia among their respective depletion groups (S2 Fig). Results depicted in Figs 2 and S2 were also analyzed and presented according to T-cell depletion regimen rather than immunization type (S3 Fig). This depletion study was also repeated with fewer immunization groups in C57BL/6J mice, and we again observed that depletion of CD4^+^ and CD8^+^ T-cells had little to no impact on the clinical disease experienced by ARPV/ZIKV-vaccinated and ZIKV-immunized mice post-challenge (Fig 3). Confirmation of T-cell depletion was done using flow cytometry (S4 Fig).

**Fig 4:**
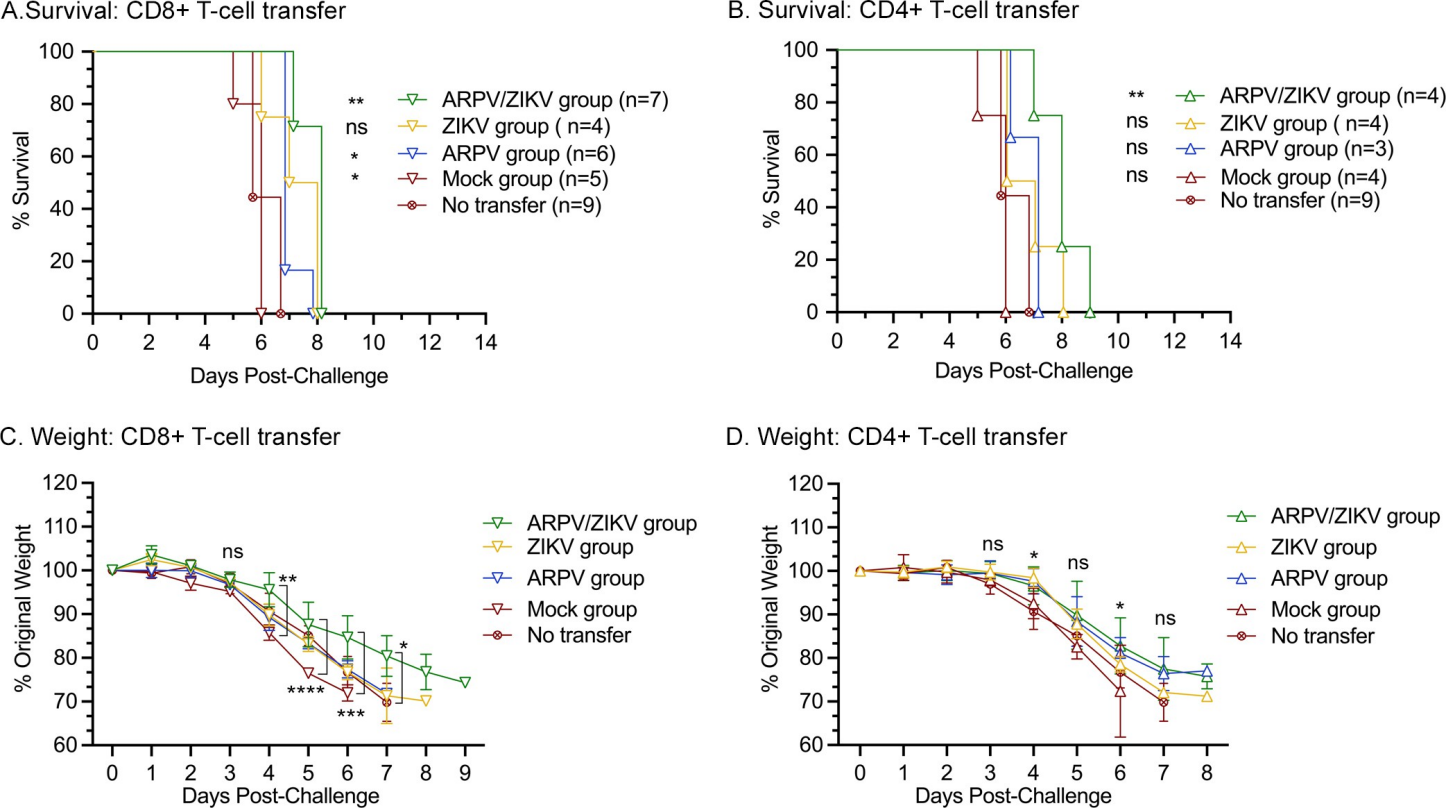
Adoptive transfer of ARPV/ZIKV-primed T-cells to naïve mice pre-challenge resulted in increased median survival times. Single-cell suspensions of CD4+ or CD8+ T-cells were prepared from the spleens of C57BL/6J mice 30 days after vaccination, then injected into naïve IFN-αβR^-/-^ mice 24 hours before challenge. Mice were then monitored for survival (a, b) and weight change (c, d). (c, d) Symbols represent mean values. Error bars indicate SD of the mean. Asterisks indicate significance compared to the “no transfer” group, unless otherwise indicated: not significant (ns), p ≤ 0.033 (*), p ≤ 0.002 (**), *p* ≤ 0.0002 (***), *p* ≤ 0.0001 (****).

To further dissect the contribution of T-cells alone in protection during a challenge, CD4^+^ and CD8^+^ T-cells were isolated from vaccinated C57BL/6J mice and transferred to naïve IFN-αβR^-/-^ mice prior to challenge (n = 4–9 recipient mice). This adoptive transfer of T-cells allowed us to examine the impact of ARPV/ZIKV-primed T-cell responses without the influence of vaccine-induced antibody responses. Overall, mice that received ARPV/ZIKV-primed CD4^+^ or CD8^+^ T-cells had a statistically significant median survival time of two days longer than control groups that received unprimed T-cells or no T-cells (Fig 4A and 4B). Mice receiving ARPV/ZIKV-primed CD8^+^ or CD4^+^ T-cells weighed more than mock transfer or no transfer mice at 4–7 dpc, or 4 and 6 dpc, respectively (Fig 4C and 4D). Collectively, these results suggest that ARPV/ZIKV-primed T-cells may contribute and play a minor role in mediating protection during challenge.

### 3.3. T-cells contribute to the development of protective immune responses after ARPV/ZIKV vaccination

**Fig 5:**
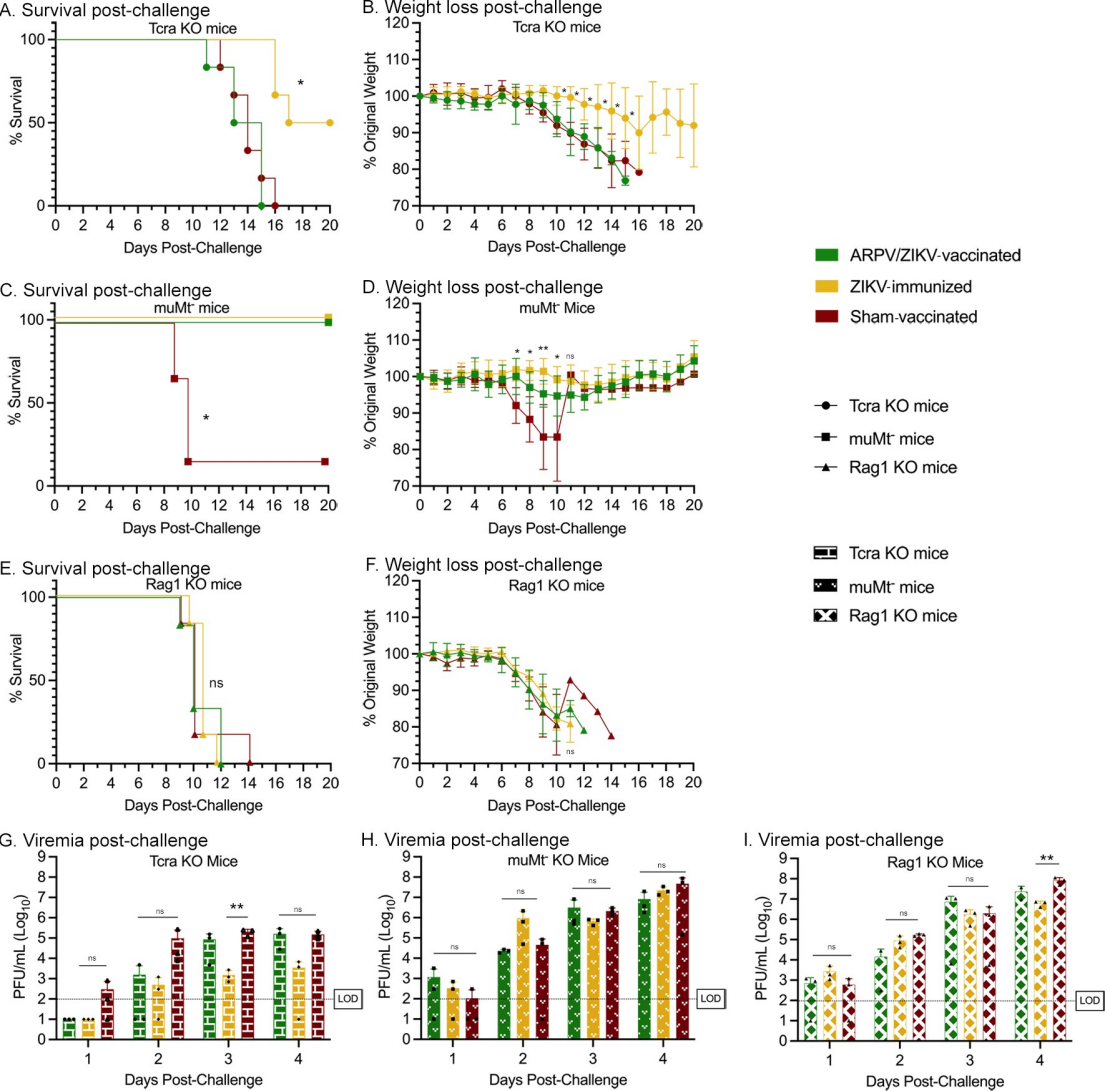
T-cells are critical for the development of an effective immune response to ARPV/ZIKV vaccination. Four-week-old mice with deficient B-cell responses (muMt^-^), deficient T-cell responses (Tcra KO), or both (Rag1 KO) were vaccinated as previously described. At 30 days post-vaccination, all mice were challenged with ZIKV. Tcra KO mice (a-b), muMt^-^ mice (c-d), and Rag1 KO mice (e-f) were then monitored for survival and weight change. (g-i) Sera were collected for 1–4 days post-challenge to quantify viremia. Dotted lines indicate the 100 pfu/mL limit of detection (LOD). (b, d, f) Symbols represent mean values. (g-i) Columns represent mean values, and symbols represent individual data points. Error bars indicate SD of the mean. Asterisks indicate significance compared to sham-vaccinated control mice, unless otherwise indicated: not significant (ns), *p* ≤ 0.033 (*), *p* ≤ 0.002 (**).

T-cells are widely multifunctional and can contribute to protective immune responses in a variety of different ways. The T-cell depletion and adoptive transfer studies above investigated the role of ARPV/ZIKV-induced T-cell responses in the protection observed at the time of challenge. However, these results do not assess the role of T-cells in aiding the development of protective immune responses during the priming and immunization phase of ARPV/ZIKV vaccination. In prior studies, *in vitro* ZIKV stimulation of ARPV/ZIKV-primed splenocytes appeared to induce significant increases in activated T-cell subpopulations, as well as IL-2 (a widely pleiotropic cytokine produced primarily by CD4^+^ T-cells, which is capable of influencing a variety of immune functions [30]) [9]. These observations led us to investigate the role of ARPV/ZIKV-induced T-cell responses in the development of immunity post vaccination. We utilized genetically modified mice to model the loss of function of various adaptive immune system branches. In particular, we used Rag1 KO mice (which lack mature B-cells and T-cells), muMt^-^ mice (which produce no mature B-cells via the *mu* gene and do not express membrane-bound IgM), and Tcra KO mice (which have dysfunctional α/β T-cell receptors and lack thymic CD4^+^CD8^-^ and CD4^-^CD8^+^ T-cells; n = 6). Mice were vaccinated with ARPV/ZIKV and challenged with ZIKV approximately four weeks post-vaccination. By 28 dpv, no mice presented ZIKV-specific nAb titers above the lower limit of detection, except for one ZIKV-immunized Tcra KO mouse which had a PRNT_50_ of 20. The lack of antibodies developed in muMt^-^ mice and Rag1 KO mice is likely due to their deficiency in B-cell responses. Although Tcra KO mice have intact B-cell responses, they likely lacked significant T-cell-dependent B-cell activation which may have significantly impaired antibody development. After ZIKV challenge, ARPV/ZIKV-vaccinated and sham-vaccinated Tcra KO mice experienced 100% mortality by 16 dpc (Fig 5A) but surviving ZIKV-immunized Tcra KO mice (50% survival) began to stabilize in weight after 17 dpc (Fig 5B). Among muMt^-^ mice, only the sham-vaccinated group experienced significant mortality and weight loss, achieving 83% mortality by 10 dpc (Fig 5C and 5D). Rag1 KO mice showed no significant differences in survival or weight loss between immunization groups (Fig 5E and 5F). ZIKV-immunized Tcra KO mice and Rag1 KO mice had significantly lower viremia compared to sham-vaccinated mice on 3 dpc and 4 dpc, respectively, whereas there were no significant differences in viremia between immunization groups in muMt^-^ mice for 1–4 dpc (Fig 5G–5J). Results depicted in Fig 5 were also analyzed and presented according to immunization group in S5 Fig. Overall, the high mortality of ARPV/ZIKV-vaccinated Tcra KO mice and the comparable severity of clinical symptoms between ARPV/ZIKV-vaccinated and mock-vaccinated mice suggest that cellular responses are critical to the development of protective immunity post ARPV/ZIKV immunization.

## 4. Discussion

Flaviviruses remain a significant global public health threat. ISFV-based vaccines may ultimately offer additional alternatives to support other traditional flavivirus vaccine platforms due to their high degree of safety, and applicability with patients for which conventional live viral vaccines may be contraindicated. Additionally, some ISFV-based vaccine candidates have demonstrated single-dose efficacy [9,14,15]. Although the underlying reasons for this have not been studied in detail, a number of factors have been suggested. For instance, viral inactivation may result in antigenic degradation. However, the natural host-restriction of the ISFV backbone means that inactivation is not required for safety. Additionally, certain ISFV-based vaccines such as ARPV/ZIKV grow to high titers in cells which facilitates highly concentrated doses during immunization. It has also been suggested that the robust immunogenicity may be partially attributed to the efficient and table delivery of the viral genome to target cells for pattern recognition receptor (PRR) detection [9]. This is supported by prior studies that demonstrate induction of mammalian innate immunity pathways by ARPV [10]. It would be interesting to dissect the mechanisms underlying the induction of immune responses for ARPV/ZIKV in comparison to ZIKV virus-like particles and lipid nanoparticles carrying ARPV/ZIKV genomic RNA to more definitively explore the influence of viral genome delivery on immunogenicity.

Previous studies show that ISFV-based vaccines can induce exceptionally high nAb titers [9,14]. However, the underlying mechanisms of immune induction and correlates of protection by this new vaccine platform, particularly regarding the role of CD4^+^ or CD8^+^ T-cell responses, remain largely unknown. For flaviviruses, neutralizing antibodies are critical for mediating protection [18,19], and cytotoxic CD8^+^ T-cells have been shown to aid in protection against ZIKV [19,21–23]. During ZIKV infection, CD4^+^ helper T-cells promote the development of nAb responses [20,31], perform cytotoxic functions and direct the immune response through cytokine-secretion [20], and may potentially protect against nervous tissue damage or provide limited protection during pregnancy [20,21]. Here, we explored T-cell-mediated responses to ISFV-based vaccines using ARPV/ZIKV as a model. Overall, we demonstrated the significant contribution of neutralizing antibodies to vaccine-induced protection through passive transfer and T-cell depletion studies. Additionally, we revealed more about the role and relative contribution of T-cell responses to vaccine-derived protection based on adoptive T-cell transfer studies and challenge studies of ARPV/ZIKV-vaccinated Tcra KO mice. We showed that T-cell responses are critical for the development of immunity post ARPV/ZIKV vaccination, and likely play a supporting role in the robust protection observed but are insufficient to provide complete protection independently.

During the passive transfer study, mice passively immunized with ARPV/ZIKV-induced immune sera were incompletely protected after challenge, despite having protective circulating nAb titers. This observation, alongside previous *ex vivo* and *in vitro* T-cell and cytokine response data [9], led to the hypothesis that ARPV/ZIKV-induced cellular immunity contributes to host protection during ZIKV challenge. Although subsequent experiments demonstrated that ARPV/ZIKV-vaccinated mice that were depleted of T-cells prior to ZIKV challenge did not experience significantly increased morbidity or mortality, adoptive transfer of ARPV/ZIKV-primed CD4^+^ and CD8^+^ T-cells to naïve mice prior to challenge resulted in a two-day increase in median survival times compared to controls. Collectively, these results may indicate that ARPV/ZIKV-induced T-cell responses contribute to protection during challenge, but that they may play a relatively minor role and be somewhat redundant when compared to the humoral immune responses. If this is the case, the partial protection after passive immunization with ARPV/ZIKV-immune sera might have been due to ARPV/ZIKV’s inability to induce antibodies against ZIKV proteins other than prM and E, such as nonstructural protein 1 (NS1). The protective ability of NS1-induced antibodies has been demonstrated with a variety of flaviviruses [32–37]. Our data would suggest that further studies should focus on exploring the complete antibody repertoire generated by ARPV/ZIKV vaccination compared to primary ZIKV infection, identify target epitopes, and explore the role of non-neutralizing antibodies in protection from disease.

In addition to investigating the role of T-cells in mediating protection during challenge, we also begin to examine the importance of T-cells prior to challenge in the context of developing ARPV/ZIKV-induced immune responses post-vaccination. ARPV/ZIKV vaccination of T-cell-deficient Tcra KO mice resulted in significantly worse clinical outcomes post-challenge compared to ZIKV immunization. Hence, T-cells likely play a critical role in developing immunity after primary exposure to ARPV/ZIKV. Interestingly, T-cell responses were sufficient for protection in both ARPV/ZIKV- and ZIKV-immunized muMt^-^ mice, despite their lack of mature B-cells and subsequent impairment of antibody responses. However, this survival of ARPV/ZIKV-vaccinated muMt^-^ mice compared to Tcra KO mice does not necessarily indicate that T-cell reactions are more important than B-cell reactions since mice deficient in T-cells likely lacked significant T-cell-dependent B-cell activation [20,31,38,39]. This is also supported by prior studies that showed CD4^+^ T-cell depletion during primary ZIKV infection negatively impacted the development of antibody responses in mice [31].

The results of the passive and adoptive transfer studies suggest that neutralizing antibodies are central to protection during challenge and that primed T-cells may play a relatively minor role. The results of the muMt^-^ mouse vaccination studies appear to be at odds with this conclusion, since 100% of ZIKV- and ARPV/ZIKV-immunized muMt^-^ mice survived post-challenge. It is important to note that direct comparisons between these studies should be made with caution given the genetic differences among the recipient mice used in each study. ZIKV NS5 does not antagonize mouse type I interferon responses, meaning that murine type I interferon responses must be depleted to render mice susceptible to infection [40–42]. The IFN-αβR^-/-^ recipient mice used in both the passive and adoptive transfer studies develop significant disease and uniformly lethal infection after ZIKV challenge, and therefore demonstrate clearer responses to protective factors [9]. In contrast, immune competent C57BL/6J mice possess intact interferon responses which must be transiently depleted using a MAR1-5A3 type I interferon antibody blockade to render these mice susceptible to ZIKV infection. However, even with MAR1-5A3 treatment, this mouse model does not typically develop lethal ZIKV disease [9,25], suggesting an incomplete blockade. muMt^-^ mice are derived from a C57BL/6J background with intact interferon responses, and although naïve muMt^-^ mice treated with MAR1-5A3 blockade do develop lethal disease (Fig 5C), this disease is less severe compared to IFN-αβR^-/-^ mice (i.e., weight loss develops slower, viremia titers are comparatively reduced in naïve muMt^-^ mice, and challenge is not uniformly lethal). It is therefore not surprising that even the modest contribution of ARPV/ZIKV-primed T-cells to protection was enough to prevent lethal disease in a MAR1-5A3-treated muMt^-^ mouse model. These results do not indicate that neutralizing antibodies are not a primary correlate of protection, but rather that primed T-cells were sufficient for protection in a mouse model that already tends to experience reduced disease burden and mortality because of an incomplete type I interferon blockade.

Overall, these studies are largely consistent with previous studies on primary and secondary ZIKV infection. Passive transfer studies of ZIKV immune sera have shown incomplete protection [31], and various CD4^+^ or CD8^+^ T-cell adoptive transfer studies have shown incomplete protection mediated by T-cells [20,21,31,38,43]. However, other studies have conflicting reports of the protective importance of adoptively transferred ZIKV-primed CD8 T+ cells [39,44]. Studies regarding T-cell-depletion during primary ZIKV infection have been similarly conflicted, but overall it seems that T-cell depletion is more likely to impact clinical outcomes or viral control during primary infection [20,21,39,43] rather than secondary infection or challenge [31]. Hence, it seems plausible that sufficiently high ZIKV-specific nAb levels are able to compensate for the absence of T-cells during secondary challenge. This is supported by studies presented here, in which the protective impact of ARPV/ZIKV-primed T-cells during challenge was only observed in the absence of circulating neutralizing antibodies (i.e., in naïve mice that received ARPV/ZIKV-primed T-cells via adoptive transfer or in ARPV/ZIKV-vaccinated muMt^-^ KO mice, but not in mice that underwent T-cell depletion). Interestingly, Nazerai et al. found that although neither CD4^+^ T-cells nor CD8^+^ T-cells were critically important in the effector phase during primary infection, CD8+ depletion did result in slightly higher viral burdens in B-cell-deficient muMt^-^ mice compared to non-depleted muMt^-^ mice [38].

These and other studies suggest that T-cell-based ZIKV immunity may serve as a backup to protective nAb responses [31,38]. Although direct comparisons between studies are complicated by differences in infection models, the results observed here are consistent with these conclusions. Particularly, the significant increase in the post-challenge clinical outcomes of ARPV/ZIKV-vaccinated Rag1 KO mice compared to vaccinated Tcra KO or muMt^-^ KO mice may support the idea of a compensatory relationship between cellular- and humoral-mediated immunity during infection. It is also possible that other immune factors may be relevant to ARPV/ZIKV vaccination. For instance, our T-cell-depletion studies and vaccination studies in B- and T-cell-deficient mice may suggest that non-T and non-B immune factors (possibly NK cells or other non-classical T-cells) together with nAbs or classical T-cell responses provide full protection upon ZIKV challenge. However, determining all the relevant immune factors will require further study.

Interestingly, ARPV/ZIKV-vaccinated muMt^-^ mice had significantly higher viremia 4 dpc than vaccinated T-cell-deficient mice, despite Tcra KO mice experiencing significantly more weight loss and death. It could be possible that Tcra KO mice had some T-cell independent B-cell activation which may have helped to control viremia during early infection. However, during late infection, B-cell-deficient mice may have been better protected due to their fully intact T-cell responses. Although further study would be needed to confirm this hypothesis, it may be consistent with previous studies that have shown that CD8^+^ T-cells may infiltrate and contribute to viral clearance in the central nervous system during neurotropic ZIKV infection [44,45], as well as studies that have shown variability in the dependence of anti-ZIKV antibody development on T-cell help [20].

In summary, knowledge of the correlates of protection and immune responses induced by ISFV-based vaccines could aid antigen design and adjuvant selection, facilitate optimization, and potentially alleviate concerns of ADE. The data shown here demonstrate that both neutralizing antibodies and T-cell responses mediate the robust protection observed, with neutralizing antibodies playing a larger role at the time of challenge and T-cells playing a significant role in the development of protective immunity post-vaccination. The immune response to ARPV/ZIKV was largely consistent with prior studies on ZIKV immunology. However, the relatively minor contribution of ARPV/ZIKV-primed CD4^+^ and CD8^+^ T-cells to protection during challenge could indicate that ISFV-based vaccine platforms may benefit from adjuvants or optimization strategies to increase T-cell responses that are protective independently of their antibody helper functions. Overall, it is likely that optimal responses to ISFV-based vaccines will require stimulation of both cell-mediated and humoral immunity. The data presented here contribute to the continued refinement of ISFV vaccine platforms, and aid in the creation of new tools for reducing the global burden of flavivirus disease.

## Supporting information

S1 Fig Neutralizing antibodies of IFN-αβR-/- mice prior to T-cell depletion. Mice were vaccinated as previously described and bled 26 days later to quantify the amount of ZIKV-specific neutralizing antibodies (nAb) in the blood. Dotted lines indicate the 20-fold and 640-fold limits of detection (LOD). Columns represent mean values, and symbols represent individual data points. Error bars indicate SD of the mean. Significance: not significant (ns), *p* ≤ 0.0001 (****).
(DOCX)

S2 Fig Data from unchallenged mice and ARPV-immunized mice described in Fig 2. (a-b) Survival post-challenge. (c-d) Weight lost post-challenge. (e-f) Viremia for 1–4 days post-challenge. Dotted lines indicate the 100 pfu/mL limit of detection (LOD). (c-d) Symbols represent mean values. (e-f) Columns represent mean values, and symbols represent individual data points. Error bars indicate SD of the mean. Not significant (ns).
(DOCX)

S3 Fig Data from the experiment described in Fig 2 was re-analyzed according to depletion group rather than immunization group. (a-e) Survival post-challenge. (f-j) Weight loss post-challenge. (k-o) Viremia post-challenge. Dotted lines indicate the 100 pfu/mL limit of detection (LOD). (f-j) Symbols represent mean values. (k-o) Columns represent mean values, and symbols represent individual data points. Error bars indicate SD of the mean. Asterisks indicate significance compared to healthy mice (unchallenged controls), unless otherwise indicated: not significant (ns), *p* ≤ 0.033 (*), *p* ≤ 0.002 (**), *p* ≤ 0.0002 (***), *p* ≤ 0.0001 (****).
(DOCX)

S4 Fig Confirmation of T-cell depletion. Groups of age-matched mice received the same T-cell depleting antibodies as experimental groups for T-cell depletion studies in (a) IFN-αβR^-/-^ mice and (b) C57BL/6J mice. At 0 days post-challenge, these age-matched groups were euthanized and their blood analyzed for circulating CD4^+^ or CD8^+^ T-cells using flow cytometry to confirm efficiency of depletion.
(DOCX)

S5 Fig Data from the experiment described in Fig 5 was re-analyzed according to immunization group. (a-c) Survival post-challenge. (d-f) Weight loss post-challenge. (g-i) Viremia post-challenge. Dotted lines indicate the 100 pfu/mL limit of detection (LOD). (d-f) Symbols represent mean values. (g-i) Columns represent mean values, and symbols represent individual data points. Error bars indicate SD of the mean. Asterisks indicate significance compared to muMt^-^ mice, unless otherwise indicated: not significant (ns), *p* ≤ 0.033 (*), *p* ≤ 0.002 (**), *p* ≤ 0.0002 (***).
(DOCX)

S1 Raw Data This file contains the raw numerical data used to construct all graphs and figures, except for flow cytometry analysis. (XLSX)

S1 File This compressed folder contains the FCS files used for flow cytometry analysis. (ZIP)
