# Supplementary material for: Humoral and T-cell-mediated responses to an insect-specific flavivirus-based Zika virus vaccine candidate
Source: PLoS Pathog. 2024 Oct 10;20(10):e1012566. doi: 10.1371/journal.ppat.1012566 (PMC11495591; doi:10.1371/journal.ppat.1012566)
Supplement: S1 Fig — Mice were vaccinated as previously described and bled 26 days later to quantify the amount of ZIKV-specific neutralizing antibodies (nAb) in the blood. Dotted lines indicate the 20-fold and 640-fold limits of detection (LOD). Columns represent mean values, and symbols represent individual data points. Error bars indicate SD of the mean. Significance: not significant (ns), p ≤ 0.0001 (****). (DOCX) [file ppat.1012566.s001.docx]

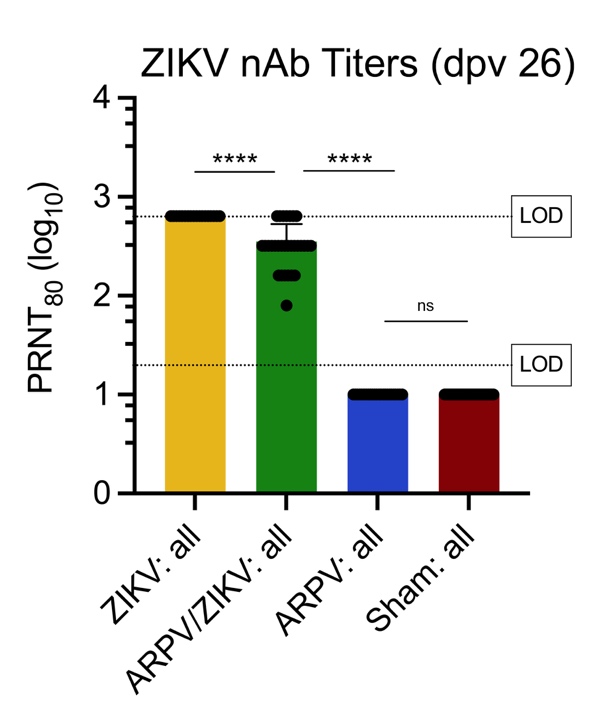


**S1 Figure: Neutralizing antibodies of IFN-αβR^-/-^ mice prior to T-cell depletion.** Mice were vaccinated as previously described and bled 26 days later to quantify the amount of ZIKV-specific neutralizing antibodies (nAb) in the blood. Dotted lines indicate the 20-fold and 640-fold limits of detection (LOD). Columns represent mean values, and symbols represent individual data points. Error bars indicate SD of the mean. Significance: not significant (ns), *p* ≤ 0.0001 (****).
