# Supplementary material for: Humoral and T-cell-mediated responses to an insect-specific flavivirus-based Zika virus vaccine candidate
Source: PLoS Pathog. 2024 Oct 10;20(10):e1012566. doi: 10.1371/journal.ppat.1012566 (PMC11495591; doi:10.1371/journal.ppat.1012566)
Supplement: S2 Fig — (a-b) Survival post-challenge. (c-d) Weight lost post-challenge. (e-f) Viremia for 1–4 days post-challenge. Dotted lines indicate the 100 pfu/mL limit of detection (LOD). (c-d) Symbols represent mean values. (e-f) Columns represent mean values, and symbols represent individual data points. Error bars indicate SD of the mean. Not significant (ns). (DOCX) [file ppat.1012566.s002.docx]

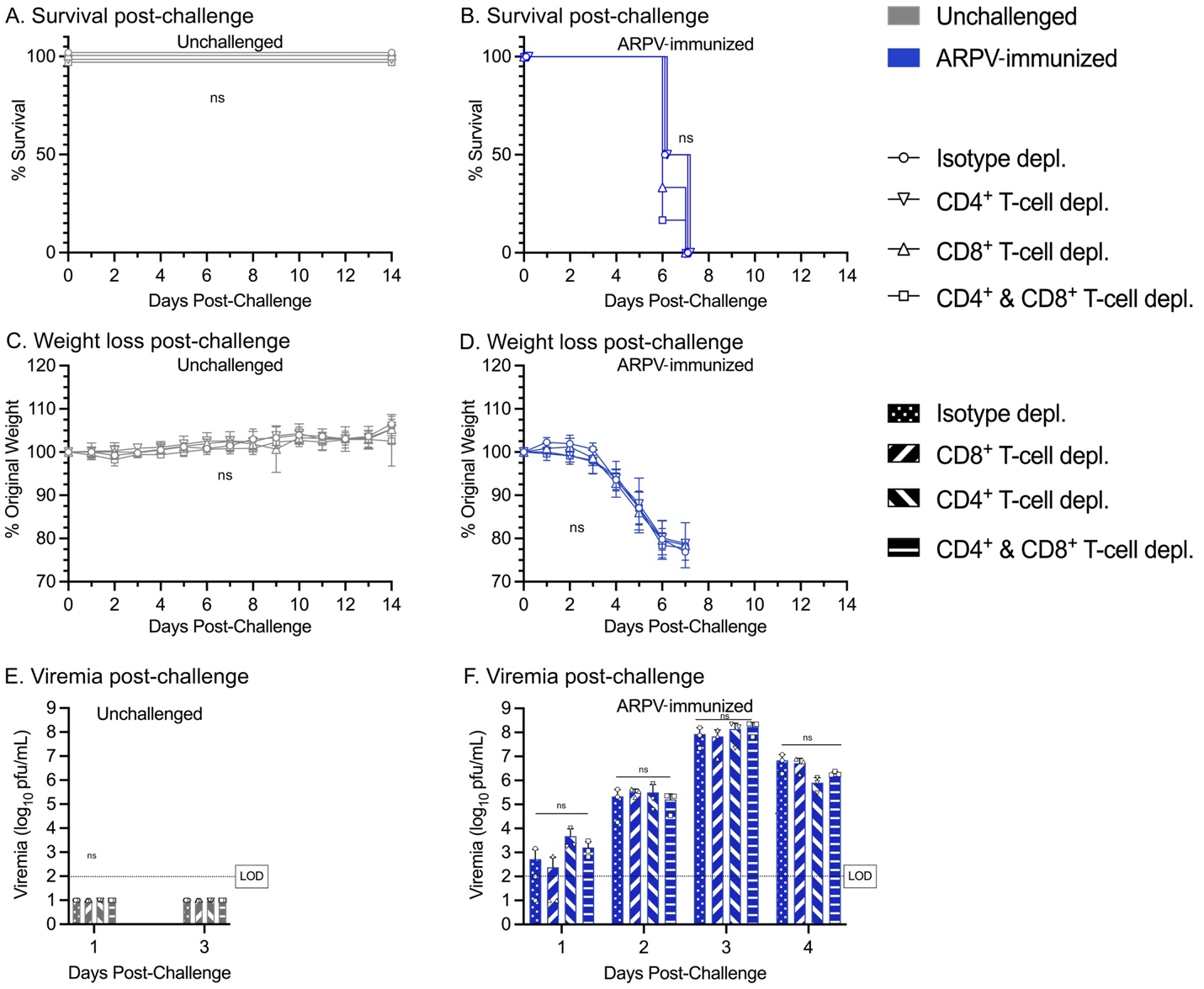


**S2 Figure: Data from unchallenged mice and ARPV-immunized mice described in Figure 2.** (a-b) Survival post-challenge. (c-d) Weight lost post-challenge. (e-f) Viremia for 1-4 days post-challenge. Dotted lines indicate the 100 pfu/mL limit of detection (LOD). (c-d) Symbols represent mean values. (e-f) Columns represent mean values, and symbols represent individual data points. Error bars indicate SD of the mean. Not significant (ns).
