# Supplementary material for: Humoral and T-cell-mediated responses to an insect-specific flavivirus-based Zika virus vaccine candidate
Source: PLoS Pathog. 2024 Oct 10;20(10):e1012566. doi: 10.1371/journal.ppat.1012566 (PMC11495591; doi:10.1371/journal.ppat.1012566)
Supplement: S3 Fig — (a-e) Survival post-challenge. (f-j) Weight loss post-challenge. (k-o) Viremia post-challenge. Dotted lines indicate the 100 pfu/mL limit of detection (LOD). (f-j) Symbols represent mean values. (k-o) Columns represent mean values, and symbols represent individual data points. Error bars indicate SD of the mean. Asterisks indicate significance compared to healthy mice (unchallenged controls), unless otherwise indicated: not significant (ns), p ≤ 0.033 (*), p ≤ 0.002 (**), p ≤ 0.0002 (***), p ≤ 0.0001 (****). (DOCX) [file ppat.1012566.s003.docx]

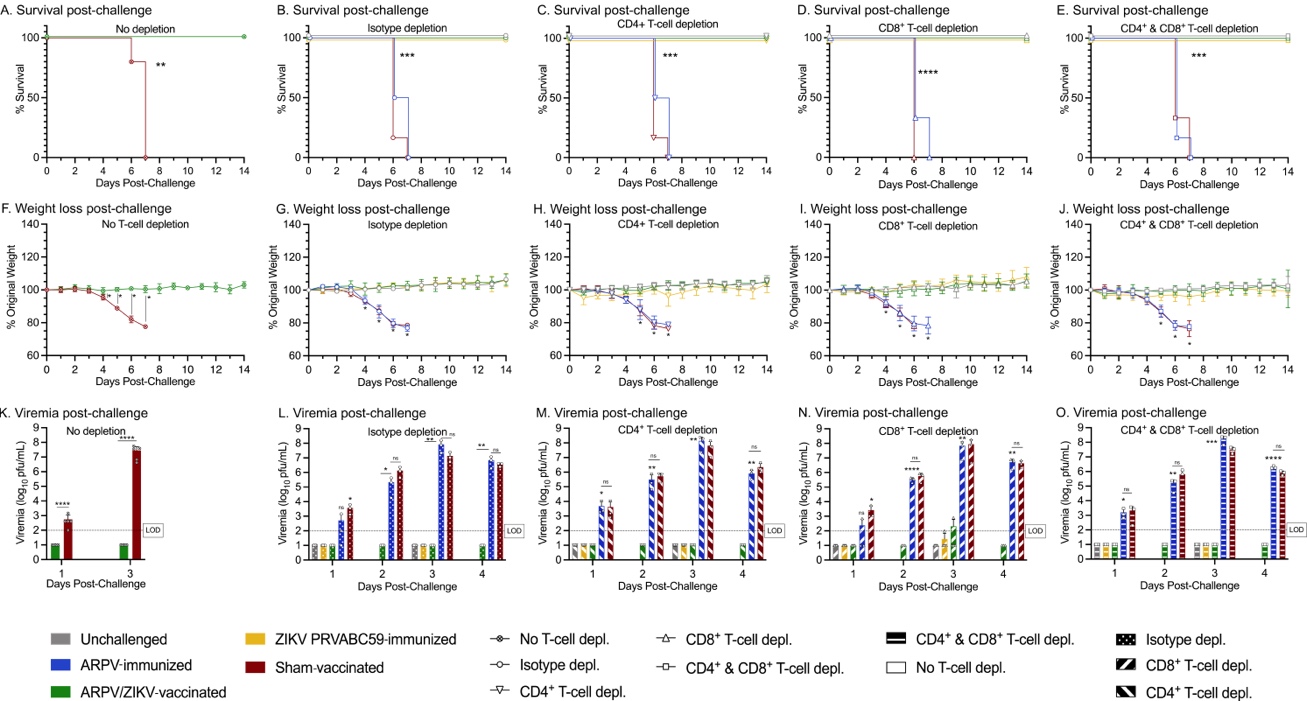


**S3 Figure: Data from the experiment described in Figure 2 was re-analyzed according to depletion group rather than immunization group.** (a-e) Survival post-challenge. (f-j) Weight loss post-challenge. (k-o) Viremia post-challenge. Dotted lines indicate the 100 pfu/mL limit of detection (LOD). (f-j) Symbols represent mean values. (k-o) Columns represent mean values, and symbols represent individual data points. Error bars indicate SD of the mean. Asterisks indicate significance compared to healthy mice (unchallenged controls), unless otherwise indicated: not significant (ns), *p* ≤ 0.033 (*), *p* ≤ 0.002 (**), *p* ≤ 0.0002 (***), *p* ≤ 0.0001 (****).
