# Supplementary material for: Humoral and T-cell-mediated responses to an insect-specific flavivirus-based Zika virus vaccine candidate
Source: PLoS Pathog. 2024 Oct 10;20(10):e1012566. doi: 10.1371/journal.ppat.1012566 (PMC11495591; doi:10.1371/journal.ppat.1012566)
Supplement: S4 Fig — Groups of age-matched mice received the same T-cell depleting antibodies as experimental groups for T-cell depletion studies in (a) IFN-αβR-/- mice and (b) C57BL/6J mice. At 0 days post-challenge, these age-matched groups were euthanized and their blood analyzed for circulating CD4+ or CD8+ T-cells using flow cytometry to confirm efficiency of depletion. (DOCX) [file ppat.1012566.s004.docx]

**
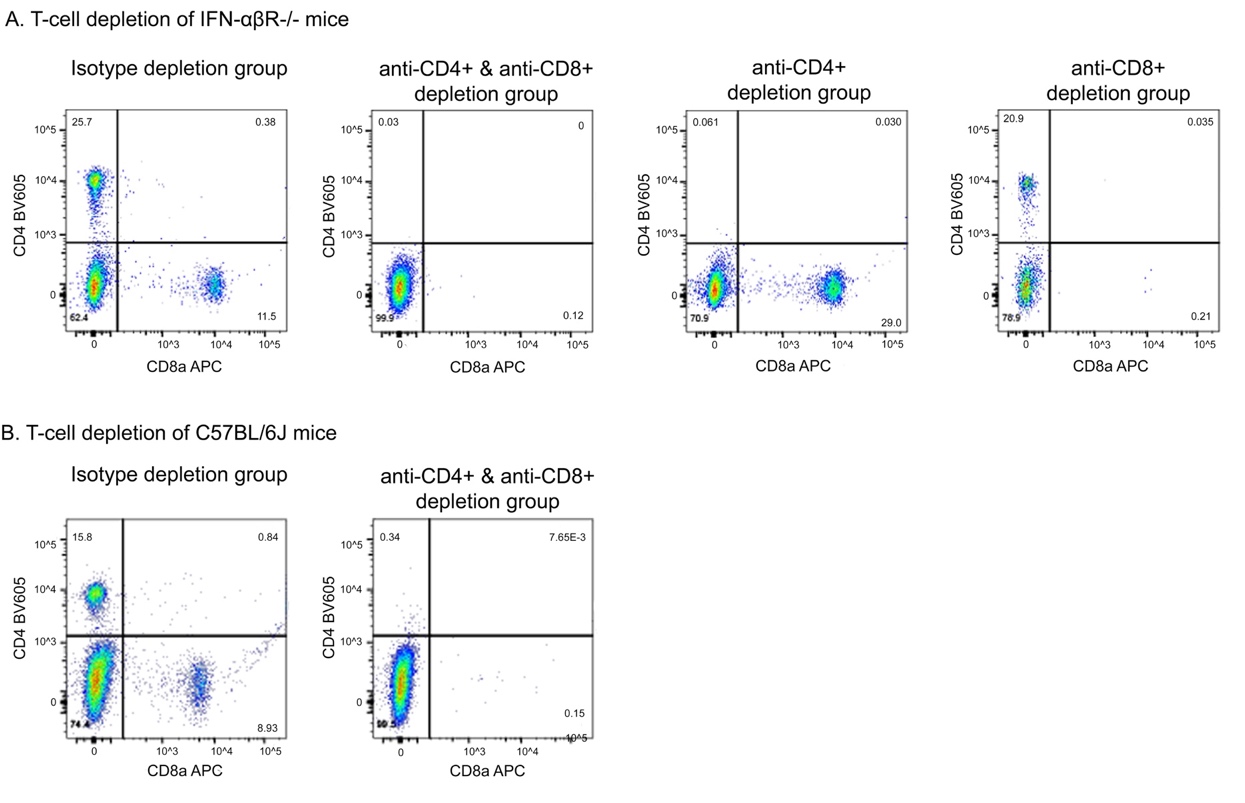
**

**S4 Figure: Confirmation of T-cell depletion.** Groups of age-matched mice received the same T-cell depleting antibodies as experimental groups for T-cell depletion studies in (a) IFN-αβR^-/-^ mice and (b) C57BL/6J mice. At 0 days post-challenge, these age-matched groups were euthanized and their blood analyzed for circulating CD4^+^ or CD8^+^ T-cells using flow cytometry to confirm efficiency of depletion.
